# 

**Published:** 1850-05

**Authors:** 


### CONTENTS

### OF NO. V.

### ORIGINAL COMMUNICATIONS.

### ESSAYS AND CASES.

Art. I.—The History, Diagnosis and Treatment of Fibrinous Coagula of the Heart. By David W. Yandell, M. D., of Louisville, Ky., Attending Physician for the Faculty at the Louisville Marine Hospital. - - - 369

Art. II.—The Bandage in the Treatment of Fractures, Ulcers, Gun-Shot Wounds, Hemorrhage, &c., &c. By Theodore S. Bell, M. D. of Louisville, Ky. - - 390

### REVIEWS.

Art. III.—A Universal Formulary: containing the methods of preparing and administering offiicinal and other medicines. The whole adapted to physicians and pharmaceutists. By R. Eglesfeld Griffith, M. D. 428

Art. IV.—The Annual of Scientific Discovery; or, Year Book of Facts in Science and Art—Exhibiting the most important discoveries and improvements in mechanics, useful arts, natural philosophy, chemistry, astronomy, meteorology, zoology, botany, mineralogy, geology, geography, antiquities. Together with a list of recent scientific publications; a classified list of patents; obituaries of eminent scientific men; an index of important papers in scientific journals, reports, etc., etc. Edited by David Wells, of the Lawrence Scientific School, Cambridge, and George Bliss, Jr. - - - - 433

Art. V.—A Practical Treatise on Inflammation of the Uterus and its Appendages, and on Ulceration and Induration of the Neck of the Uterus. By James Henry Ben-

NET, M. D., M. R. C. P., Physician-Accoucheur to the Western General Dispensatory; formerly House-Physician (by concours) to the hospitals Saint Louis, Notre Dame, La Pitie, and La Salpetriere, Paris. - 440

SELECTIONS FROM AMERICAN AND FOREIGN JOURNALS.

New Instrument for Operating on Hemorrhoids, - - 441
Impropriety of Frequent Operative Interference in Midwifery, - 441
New Remedy for Leucorrhea, - - - - 442
On Opacities of the Cornea, - - - - - 443
Case of Malignant Tumor, - - - - 448

ORIGINAL INTELLIGENCE.

A Professorial Thersites, - - - - - 453
Prof. Bigelow's Intodoctory Lecture—Clinical Lectures, - 455
Veterinary Medicine, - - - - - 458
Demonstrative Midwifery—The Buffalo Medical Journal, - 459

